# The efficacy of platelet-rich fibrin in alveolar ridge preservation: a systematic review and meta-analysis of randomized controlled trials

**DOI:** 10.3389/fmed.2026.1753189

**Published:** 2026-02-19

**Authors:** Jiahui Yan, Kexin Lu

**Affiliations:** Department of Dentistry, The Second Affiliated Hospital of Zhejiang Chinese Medical University, Hangzhou, China

**Keywords:** alveolar ridge preservation, bone regeneration, meta-analysis, platelet-rich fibrin, randomized controlled trial

## Abstract

**Objectives:**

The aim of this study was to systematically evaluate the independent efficacy of platelet-rich fibrin (PRF) as a sole grafting material in alveolar ridge preservation and to dynamically delineate the trajectory of PRF’s effects on alveolar ridge morphology and new bone formation across various healing stages.

**Methods:**

A comprehensive search was conducted across four databases (PubMed, Embase, the Cochrane Library, and Web of Science) for pertinent records from their establishment until November 2025. The study encompassed randomized controlled trials (RCTs) that evaluated alveolar ridge preservation utilizing PRF alone in comparison to spontaneous healing. Two investigators independently conducted literature screening, data extraction, and methodological quality evaluation, employing the Cochrane Risk of Bias instrument (ROB-2) for the latter. The certainty of the evidence for each outcome was assessed using the GRADE framework. The meta-analysis utilized RevMan version 5.3, while publication bias was evaluated by Egger’s test in Stata 18.

**Results:**

A total of eighteen studies were incorporated into the analysis. Compared to the spontaneous healing group, the PRF group showed significantly smaller losses in alveolar bone height at 3 months (*p* = 0.004; 95% CI = −1.59 to −0.31), 4 months (*p* = 0.002; 95% CI = −0.93 to −0.20), and 6 months (*p* = 0.03; 95% CI = −0.53 to −0.03). The use of PRF resulted in a significantly lower reduction in alveolar bone width at 2 months (*p* = 0.03; 95% CI = −1.18 to −0.05) and 3 months. The percentage of new bone formation in the PRF group was significantly greater than that in the spontaneous healing group at both 3 months (*p* = 0.0008; 95% CI: 4.70–18.02) and 4 months (*p* = 0.010; 95% CI: 4.14–29.81). High-speed centrifugation was associated with significantly greater new bone formation than standard protocols (*p* = 0.02), while effects on dimensional preservation did not differ significantly between protocols.

**Conclusion:**

PRF appears to mitigate alveolar bone resorption following tooth extraction and may enhance new bone formation during the healing process. The osteogenic effect of PRF may be optimized by high-speed centrifugation protocols. As a secure autologous biomaterial, it is a promising option for preserving alveolar bone and enhancing circumstances for eventual implant restoration.

**Systematic review registration:**

https://www.crd.york.ac.uk/PROSPERO/view/CRD420251183872, identifier PROSPERO (CRD420251183872).

## 1 Introduction

The alveolar ridge experiences several unavoidable physiological changes after tooth extraction, chief among them being a notable reduction in height and width (1). It indicates that within the initial 6 months post-extraction, the alveolar ridge width may diminish by an average of 3.0–4.5 mm, and the height by an average of 1.0–1.5 mm, predominantly affecting the buccal bone plate (2, 3). This gradual loss of bone frequently leads to an unacceptable morphology of the alveolar ridge, which can have a substantial impact on implant placement, the aesthetics of restorations, and even the complexity of surgery (4).

To minimize alveolar ridge resorption following tooth extraction and establish optimal conditions for dental implants, alveolar ridge preservation techniques have been developed. This technique entails the placement of diverse bone graft materials or biomaterials into the extraction socket concurrently to support soft tissues, stabilize blood clots, and facilitate bone regeneration (5). Conventional materials for alveolar ridge preservation comprise xenogeneic bone grafts and collagen plugs. While these materials exhibit favorable clinical results, they are associated with drawbacks, including high costs, restricted availability, and possible immunological risk. As a second-generation platelet concentrate, PRF is recognized as an optimal biomaterial due to its straightforward preparation method that does not necessitate anticoagulants or external biochemical additives. It is characterized by a high concentration of growth factors, including platelet-derived growth factor (PDGF), transforming growth factor-β (TGF-β), and vascular endothelial growth factor (VEGF), along with a three-dimensional fibrin network structure (6, 7). These growth factors can actively control inflammatory responses, speed up neovascularization, and promote the growth and differentiation of osteoblasts and fibroblasts. The results stops bone resorption and helps new bone form. Thus, it is widely employed in dentistry to promote the healing of extraction sockets, assist in maxillary sinus enlargement, and aid periodontal regeneration (8, 9).

Currently, multiple randomized controlled trials have been conducted to assess the efficacy of PRF in alveolar ridge preservation. These studies demonstrate significant variability in sample size, intervention protocols, and other factors, which complicates the ability of individual studies to yield universally applicable high-level evidence. Existing systematic reviews present two key limitations that our study seeks to address. First, they have primarily focused on evaluating PRF in conjunction with other bone graft materials, thus providing evidence for its adjunctive role rather than its intrinsic efficacy as a sole grafting agent. The effect of PRF alone remains less clearly quantified. Second, and more critically, prior syntheses have typically reported pooled effects across mixed follow-up periods or at single time points. This “static” analytical approach cannot answer whether the benefits of PRF are immediate or progressive, or how its spatial maintenance capacity versus its bioactive osteogenic stimulation evolves over the healing period. Therefore, this systematic review and meta-analysis was designed with two novel aims that distinguish it from prior work: (1) to provide a pure estimate of the efficacy of PRF used alone for alveolar ridge preservation by synthesizing only RCTs where PRF was the sole socket-filling material; and (2) to employ a pre-planned, longitudinal analytical framework via time-stratified meta-analysis (at 2, 3, 4, and 6 months) to elucidate the dynamic trajectory of PRF’s effects on alveolar ridge height, width, and new bone formation. This approach moves beyond asking if PRF works to investigate how its effects manifest and change over time, offering clinicians evidence-based guidance on optimal observation windows and a more nuanced understanding of its mechanism of action in socket healing.

## 2 Materials and methods

### 2.1 Protocol registration and guidelines

This systematic review and meta-analysis adhered rigorously to the PRISMA (Preferred Reporting Items for Systematic Reviews and Meta-Analyses) standards (10). The study protocol is filed on the PROSPERO platform (Registration Number: CRD420251183872).

### 2.2 Search strategy

The databases PubMed, Embase, Cochrane Library, and Web of Science were methodically queried. The timeframe extends from indexing until November 2025. Searches utilized a blend of subject headings and free-text terminology. The primary search terms encompassed “platelet-rich fibrin,” “fibrin, platelet-rich,” “platelet rich fibrin,” “leukocyte- and platelet-rich fibrin,” “leukocyte and platelet-rich fibrin,” “l-PRF,” “leukocyte PRF,” “p-PRF,” “pure platelet-rich fibrin,” “pure PRF,” “thrombocyte-rich fibrin,” “socket preservation,” “ridge preservation,” “alveolar ridge preservation,” “socket grafting,” and “post-extraction grafting” (Supplementary Table S1).

### 2.3 Eligibility criteria

The research question has been formulated based on the PICOS framework. (P) Participants: systemically healthy adults aged 18 years and older who require tooth extraction for various indications and are undergoing either alveolar ridge preservation or spontaneous healing. (I) Intervention: post-extraction placement of PRF in the socket. (C) Control: natural healing of the extraction socket accompanied by the formation of a blood clot. (O) Outcomes: alveolar ridge height, alveolar bone width, and the percentage of new bone formation, quantified as the new bone volume fraction (BV/TV) via histomorphometric analysis. (S) Randomized controlled trials.

Inclusion criteria: (1) data must report at least one relevant outcome measure; (2) follow-up duration of at least 1 month. Exclusion criteria: (1) animal studies, *in vitro* research, case reports, or reviews; (2) PRF used in conjunction with other bone graft materials or biological agents; (3) studies published solely as abstracts, conference papers, or lacking full-text availability.

### 2.4 Data collection and extraction process

Two researchers conducted independent literature searches and included eligible studies according to established criteria. Any discrepancies were resolved by consensus or a third reviewer. Data were extracted from each study, including authors, country, sample size, age, gender, outcomes, assessment tools, follow-up time, PRF preparation method, and socket location.

### 2.5 Quality evaluation and risk of bias

The Cochrane ROB-2 tool was employed to evaluate risk of bias, focusing on the following components: (1) randomization method; (2) allocation concealment; (3) blinding of participants and personnel; (4) blinding of outcome assessment; (5) presence of incomplete outcome data; (6) selective reporting of outcomes; (7) other potential sources of bias.

### 2.6 Statistical analysis

A meta-analysis was performed utilizing RevMan 5.3 software. The mean difference (MD) and its 95% confidence interval (CI) were employed as the effect size for continuous variables. *I*^2^ served as a measure of heterogeneity: values below 50% indicated acceptable heterogeneity, warranting the use of a fixed-effect model; values exceeding 50% prompted an investigation into sources of heterogeneity, leading to the application of a random-effects model. Funnel plots were employed to evaluate publication bias, and Egger’s test was performed using Stata 18 software.

We acknowledge that different PRF preparation protocols may yield matrices with distinct biological properties and release kinetics of growth factors, which could significantly influence clinical outcomes and contribute to heterogeneity. Therefore, we planned to conduct subgroup analysis based on different PRF preparation protocols (centrifugation speed and centrifugation time). In accordance with the Cochrane Handbook for Systematic Reviews of Interventions, when paired outcome data (e.g., within-patient mean differences and their standard errors) were not reported in the split-mouth studies, we utilized the group-level summary statistics (means and standard deviations) presented for the intervention and control groups.

### 2.7 Assessment of the certainty of the evidence

The certainty of the evidence for each primary outcome (reduction in alveolar ridge height, reduction in alveolar ridge width, and percentage of new bone formation) was assessed using the GRADE (Grading of Recommendations, Assessment, Development, and Evaluations) approach. Two review authors (J. Y. and K. L.) independently rated the certainty of evidence. Discrepancies were resolved through discussion.

The initial certainty of evidence for all outcomes was ‘high,’ as they originated from randomized controlled trials. The certainty was then downgraded based on the following five factors: risk of bias, inconsistency, indirectness, imprecision, and publication bias. The final certainty was categorized into four levels: high, moderate, low, or very low.

## 3 Results

### 3.1 Records obtained during the search process

**Figure 1:**
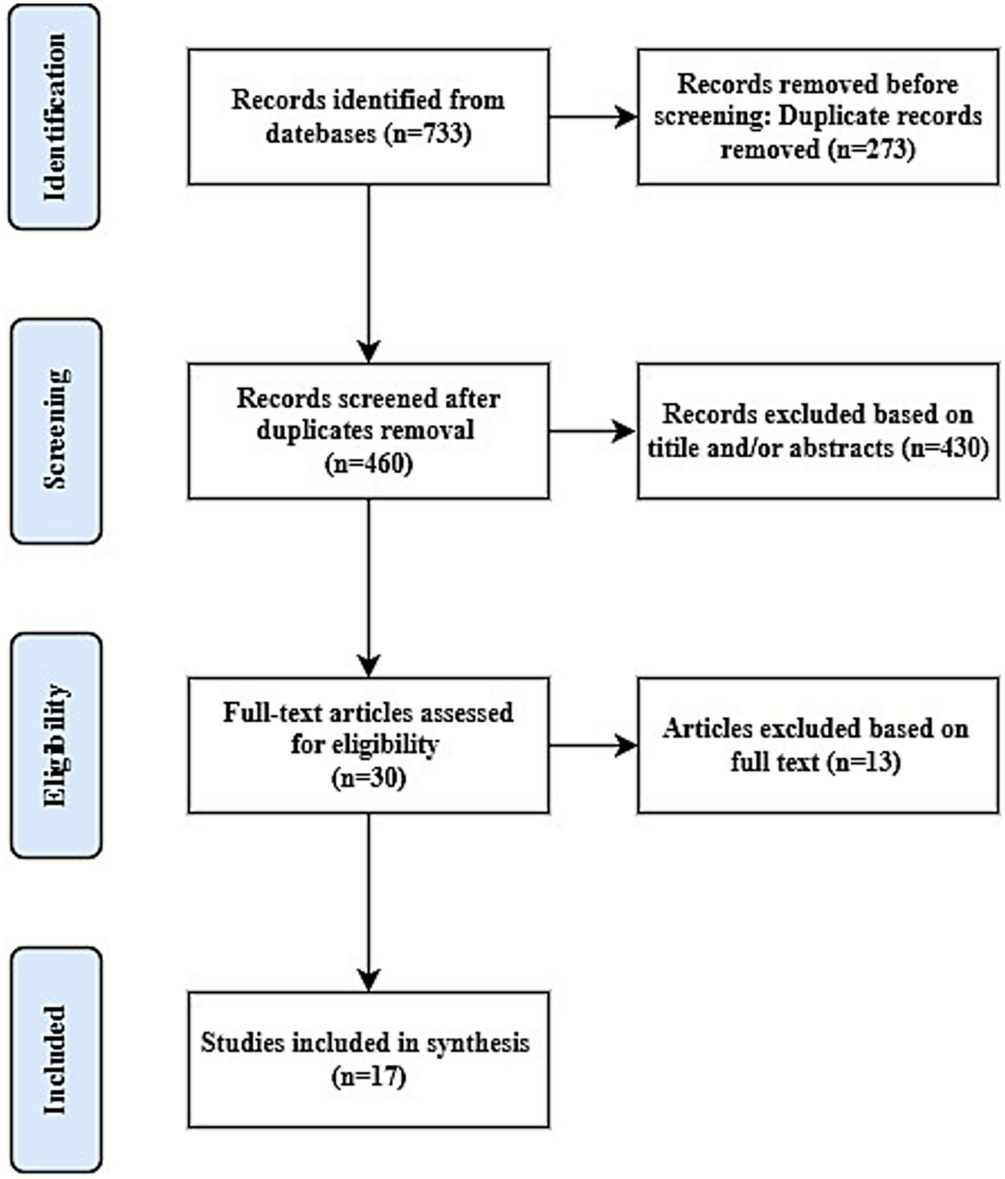
PRISMA flow diagram of study selection.

The literature screening process is detailed in the PRISMA flow diagram (Figure 1). The inquiry produced 733 publications. Following the elimination of 273 duplicates, 460 articles were subjected to preliminary screening based on titles and abstracts. The procedure led to the elimination of 430 review papers, case series/reports, animal studies, and other publications. In total, 30 full-text papers were assessed, of which 13 were excluded following the comprehensive assessment. This meta-analysis and systematic review encompassed 17 studies for quantitative evaluation.

### 3.2 Study characteristics

The key characteristics of the 17 included RCTs are summarized in Supplementary Table S2. The sample sizes varied considerably, ranging from 10 patients with 36 sockets to 90 patients. The mean age of participants spanned from approximately 34 to 58 years. The extraction sites included anterior teeth, premolars, and molars. A critical source of variation was the PRF preparation protocol: the standard leukocyte-PRF (L-PRF) protocol (2,700 rpm, 12 min) was used in twelve studies (11–22), the high-speed protocol (3,000 rpm, 10 min) in four studies (21, 23–25), and the low-speed Advanced-PRF (A-PRF) protocol (1,300 rpm, 14 min or 8 min) in one study (26). The primary outcomes consistently focused on alveolar ridge dimensional changes (height and width) and the percentage of new bone formation. These were assessed primarily using cone-beam computed tomography (CBCT) or study models (11–24, 26) and histomorphometric analysis (14, 15, 20, 21, 25–27), respectively. Follow-up assessment times were predominantly at 2, 3, 4, and 6 months postoperatively.

The main findings from each included study are presented in Table 1. Certain studies demonstrated statistically significant benefits of PRF in preserving ridge height or width at specified time intervals (12–14), whereas others noted improvements in new bone formation (14, 15, 25, 26). However, some studies found no statistically significant differences between groups for certain outcomes (11, 16, 18, 20, 24, 27), highlighting the heterogeneity in study results. These individual findings point out the need for the subsequent quantitative synthesis to derive an overall estimate of PRF’s efficacy.

**Table 1:** Main findings of included studies.

Author (year)	Main findings
Abad et al. (11) (2023)	The L-PRF group showed a trend toward less buccal vertical bone loss, but the difference was not statistically significant. Similarly, horizontal width loss decreased at levels 3 mm and 5 mm apical to the crest in both groups, with no significant intergroup differences at these levels.
Aldommari et al. (12) (2025)	L-PRF significantly reduced horizontal alveolar ridge width loss at 7 mm and 10 mm apical to the CEJ, though not at 4 mm. It also better preserved vertical bone height, especially on the buccal aspect, compared to spontaneous healing.
Alzahrani et al. (23) (2017)	The PRF group demonstrated a significantly smaller loss in alveolar ridge width, with the difference being statistically significant at both 4 and 8 weeks.
Badakhshan et al. (13) (2020)	L-PRF significantly reduced bone resorption in width (at crest, 1 mm, and 3 mm levels) and height (mesial/distal, buccal/palatal) compared to controls.
Canellas et al. (14) (2020)	L-PRF significantly reduced horizontal bone resorption (at 1 mm and 3 mm below the crest) and limited buccal vertical bone loss compared to spontaneous healing while promoting higher new bone formation.
Castro et al. (15) (2021)	L-PRF did not significantly reduce horizontal or vertical alveolar ridge resorption following multiple tooth extractions but showed statistically superior socket fill and greater new bone formation.
Girish et al. (24) (2018)	There was no statistically significant difference in ridge resorption or radiographic bone fill between the PRF group and the control group at 6 months post-extraction.
Mousav et al. (16) (2024)	L-PRF did not significantly reduce vertical ridge resorption or enhance new bone formation and resulted in greater horizontal bone loss in the coronal socket compared to natural healing.
Niedzielska et al. (17) (2022)	PRF enhanced initial soft tissue healing and diminished ridge resorption, resulting in increased bone density formation at 6 months.
Rodrigues et al. (18) (2023)	PRF has limited effectiveness in preserving three-dimensional alveolar bone volume, and its bone preservation efficacy shows no significant difference compared to natural healing.
Temmerman et al. (19) (2016)	L-PRF used as a socket filling material significantly reduces vertical and horizontal ridge resorption, enhances socket bone fill, and reduces postoperative pain at 3 months after extraction.
Zhang et al. (20) (2018)	PRF alone enhances soft tissue healing and new bone formation but does not significantly preserve ridge height or width compared to natural healing.
Clark et al. (26) (2018)	A-PRF most effectively minimized ridge height loss and produced the highest vital bone formation (46 ± 18%), significantly greater than FDBA alone (29 ± 14%).
Ivanova et al. (21) (2021)	Both PRF alone and FDBA+PRF significantly outperformed the control in vital bone formation and produced less connective tissue. No significant difference in vital bone formation was observed between the PRF group and the FDBA+PRF group.
Hauser et al. (22) (2012)	PRF (flapless) improved new bone microstructure, intrinsic quality, and ridge width preservation. PRF with a flap negated these benefits, performing similarly to simple extraction.
Aliyev et al. (25) (2025)	L-PRF showed higher new bone formation compared to the control and significantly increased ALP and PCNA expression. L-PRF resulted in the least postoperative pain and gingival swelling, with excellent healing.
Areewong et al. (27) (2019)	The new bone formation ratio was slightly higher in the PRF group compared to the control group. The difference in new bone formation ratio between the two groups was not statistically significant.

### 3.3 Results of bias risk assessment

**Figure 2:**
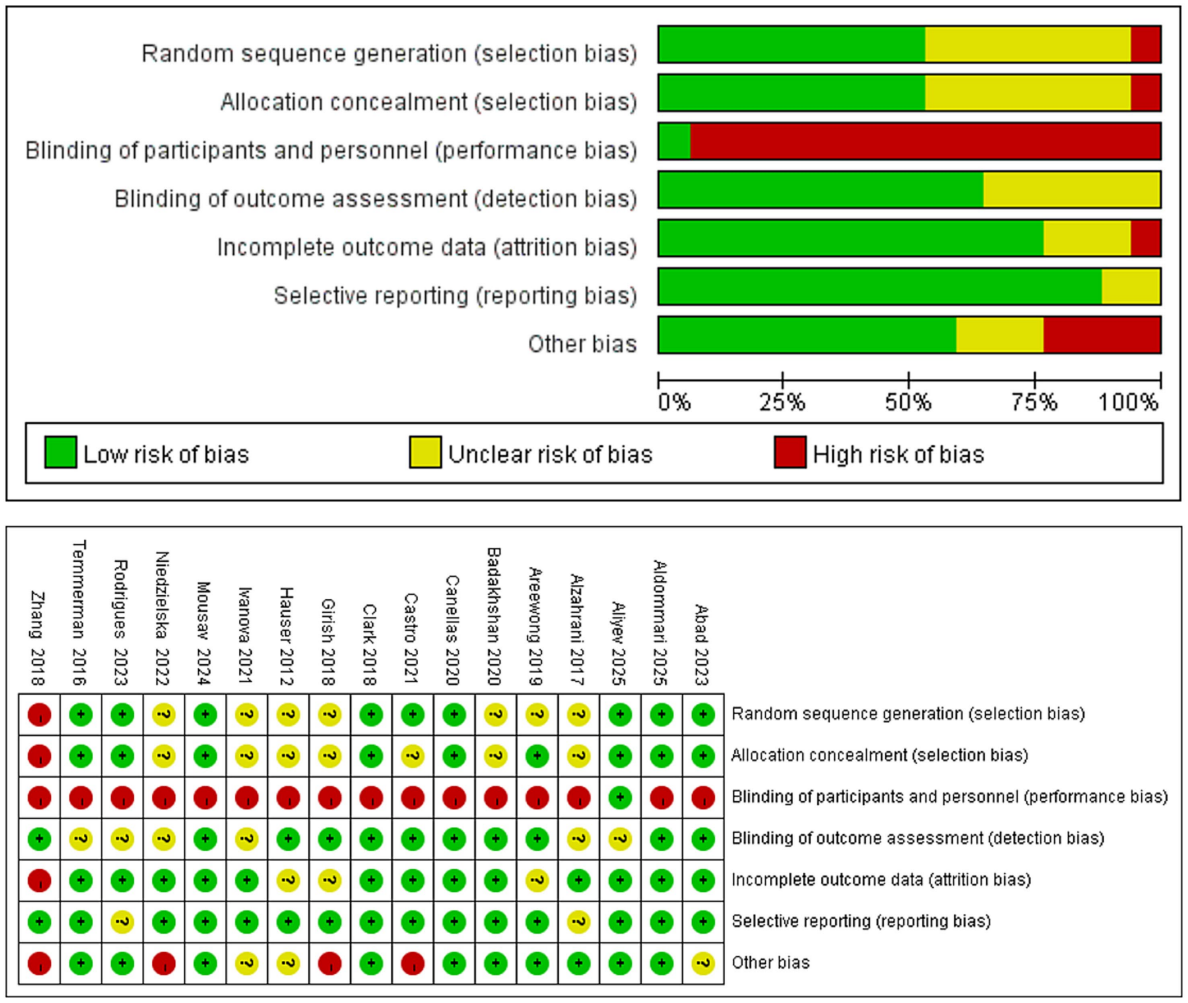
Risk of bias graph and summary for the included studies.

The risk of bias assessment for the 17 included studies indicated a profile characterized by low risk in terms of selective reporting and completeness of outcome data, while performance bias was prevalent due to the inherent nature of the PRF intervention (Figure 2). Approximately half of the studies adequately described random sequence generation (11, 12, 14–16, 18, 19, 25, 26) and allocation concealment (11, 12, 14, 16, 19, 25–27). 16 were classified as having a “high risk” of bias regarding blinding for both investigators and subjects, with only one exception (25). The blinding of outcome assessors was stated openly in the majority of trials (11–16, 20, 22, 24, 26, 27). All studies, except one (20), which was identified as high-risk due to a significant loss of follow-up and a lack of intention-to-treat analysis, exhibited strong data integrity. The risk of selective reporting was minimal. In relation to other biases, most studies indicated no significant risk, whereas eight studies presented concerns, including baseline imbalances or methodological limitations (11, 15, 17, 18, 20–22, 24). While performance bias was widespread, its prevalence is an inherent issue in surgical trials comparing an intervention (PRF) to passive healing. Importantly, the two main findings of this meta-analysis are quantitative, objective measurements: the histomorphometric new bone percentage and the alveolar ridge dimensions as determined by CBCT. The assessment of these outcomes was blinded in the majority of included studies, decreasing the likelihood of measurement bias. As a result, it was determined that the overall risk of bias for these objective endpoints was insufficient to render the results incorrect.

### 3.4 Meta-analysis results

#### 3.4.1 Alveolar ridge height

**Figure 3:**
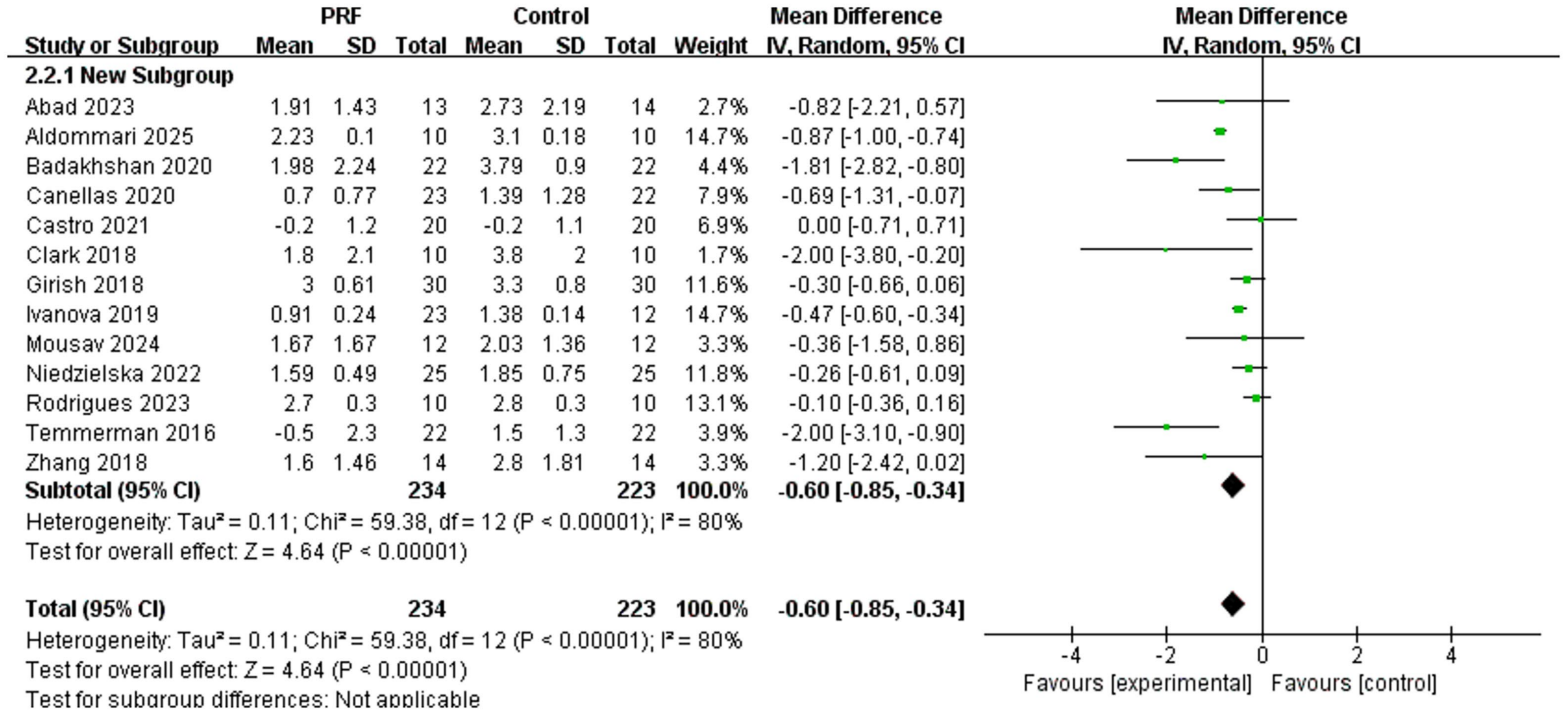
Forest plot for the meta-analysis of alveolar ridge height reduction: PRF group versus control group.

Thirteen studies documented alterations in alveolar ridge height (11–21, 24, 26), encompassing 234 cases in the PRF group and 223 cases in the control group. The pooled results indicated a statistically significant reduction in alveolar ridge height loss in the PRF group compared to the control group (*I*^2^ = 80%; *p* < 0.00001; MD = −0.60; 95% CI = −0.85 to −0.34) (Figure 3). However, the substantial heterogeneity (*I*^2^ = 80%) indicates considerable variation in the magnitude of this effect across studies.

**Figure 4:**
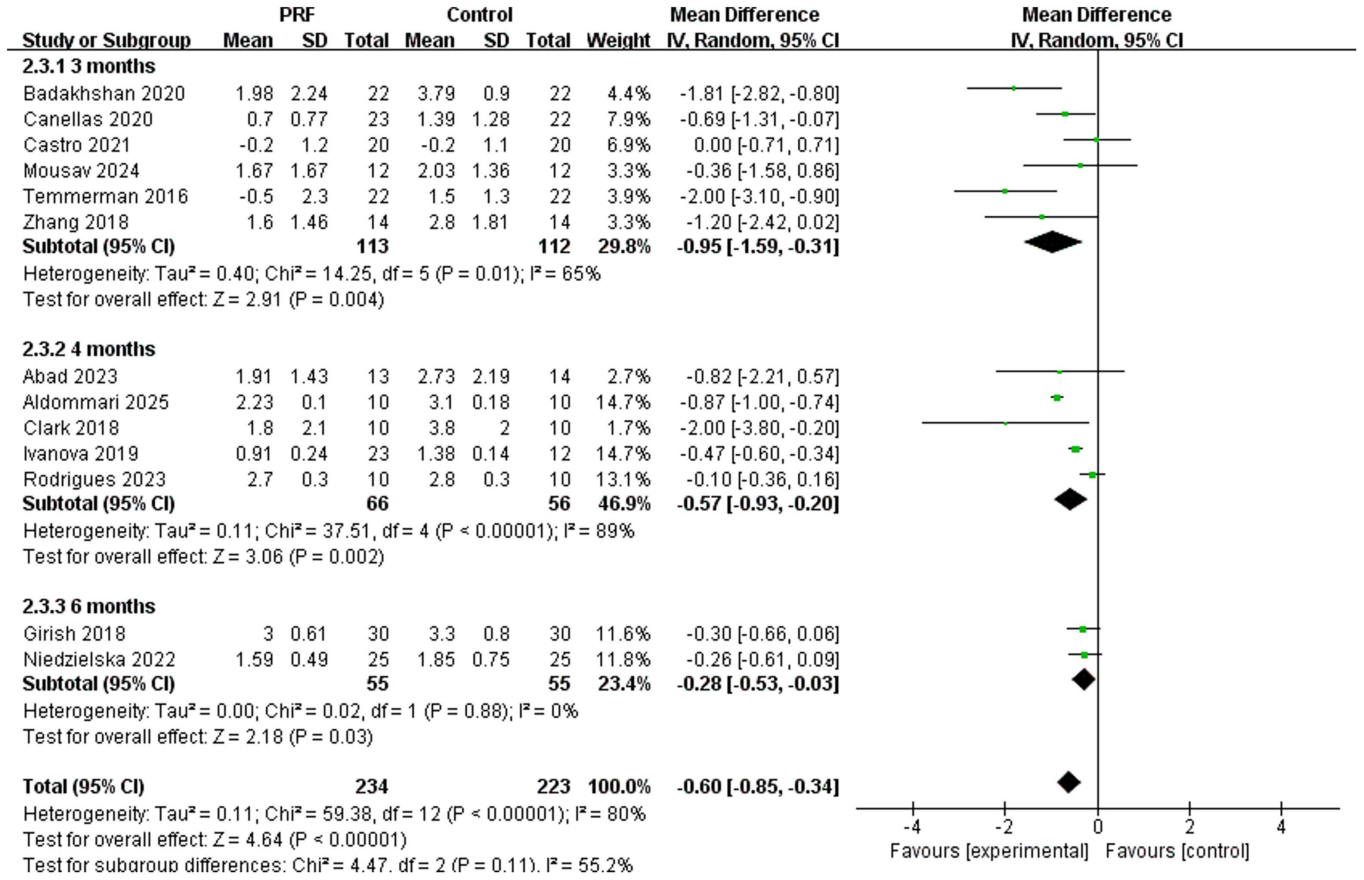
Forest plot for the subgroup meta-analysis of alveolar ridge height reduction at different postoperative time points (3, 4, and 6 months): PRF group versus control group.

Subgroup analysis assessed the reduction in alveolar bone height at various time points (Figure 4). At 3 months, there was a significant reduction in bone height with PRF (*I*^2^ = 65%; *p* = 0.004; MD = −0.95; 95% CI = −1.59 to −0.31) (13–16, 19, 20, 26). Similar significant results were observed at 4 months (*I*^2^ = 89%; *p* = 0.002; MD = −0.57; 95% CI = −0.93 to −0.20) (11, 12, 18, 21, 26) and at 6 months (*I*^2^ = 0%; *p* = 0.03; MD = −0.28; 95% CI = −0.53 to −0.03) (17, 24). There was no statistically significant difference in the preservation effect of PRF on alveolar bone height at different time periods (*p* = 0.11).

#### 3.4.2 Alveolar ridge width

**Figure 5:**
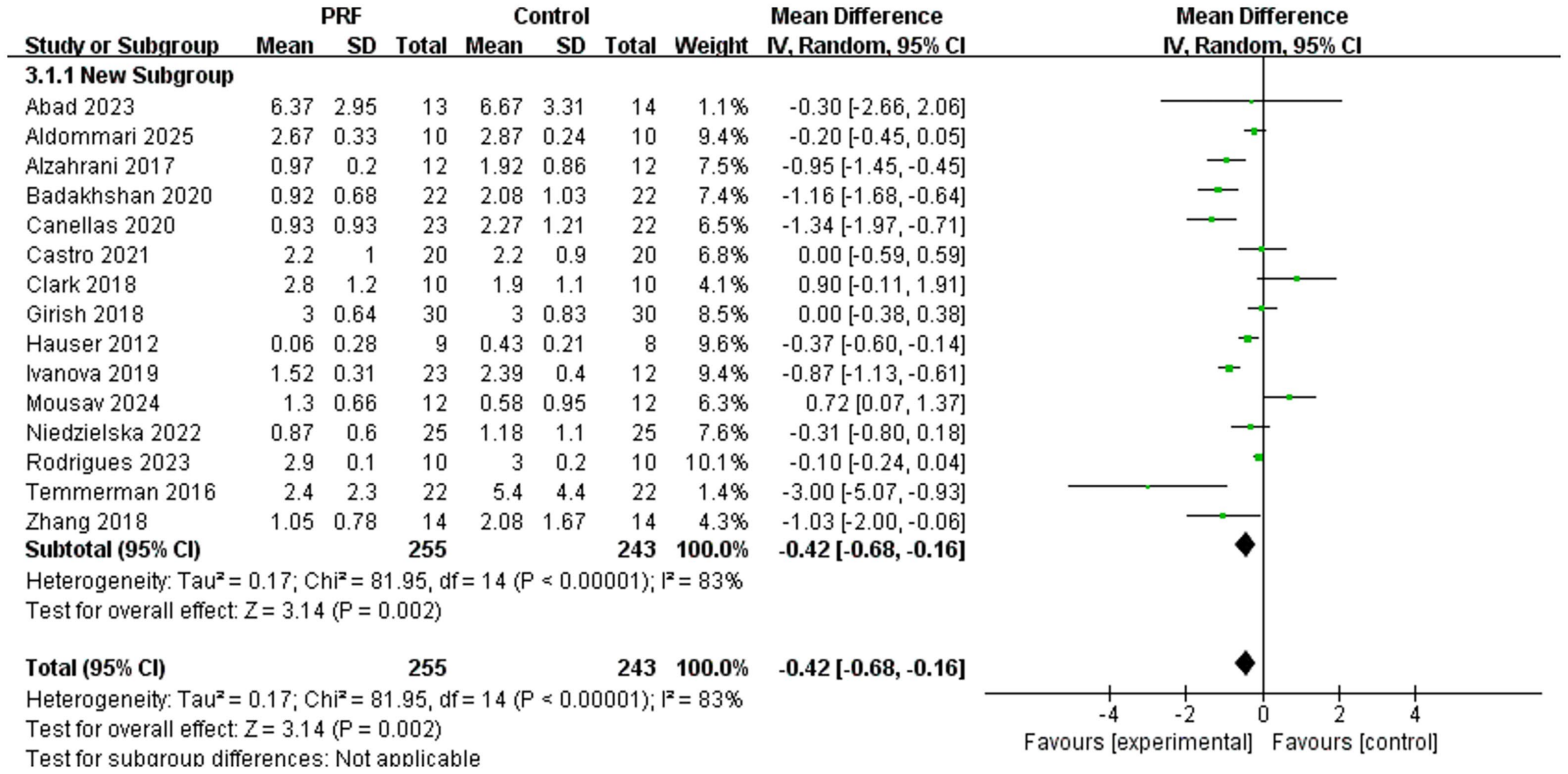
Forest plot for the meta-analysis of alveolar ridge width reduction: PRF group versus control group.

Sixteen studies documented alterations in alveolar ridge width, encompassing 255 cases in the PRF group and 243 cases in the control group (11–24, 26). The pooled analysis suggested a reduction in alveolar ridge width loss with PRF compared to the control (*I*^2^ = 83%; *p* = 0.002; MD = −0.42; 95% CI = −0.68 to −0.16) (Figure 5).

**Figure 6:**
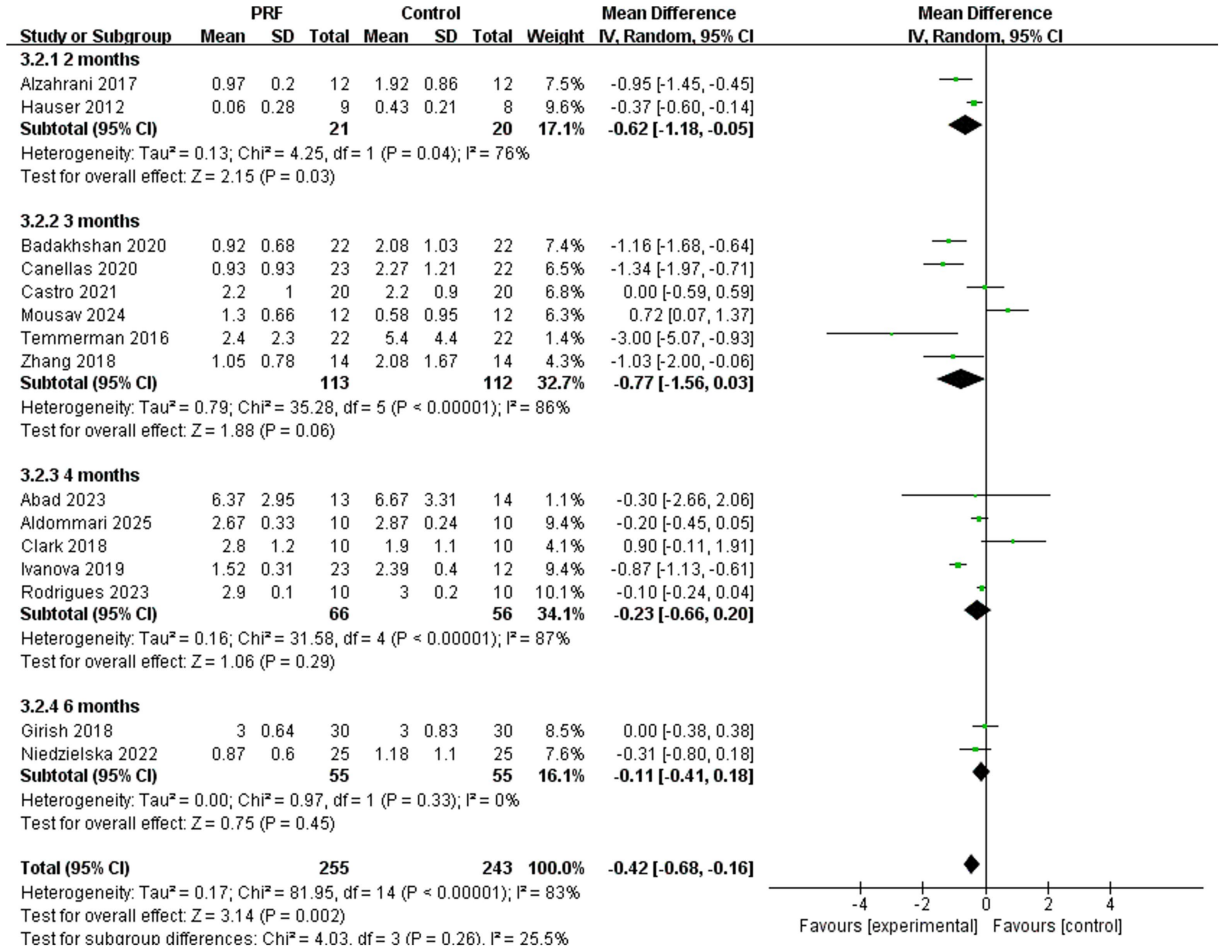
Forest plot for the subgroup meta-analysis of alveolar ridge width reduction at different postoperative time points (2, 3, 4, and 6 months): PRF group versus control group.

Subgroup analyses evaluated the loss of alveolar bone width at various time intervals (Figure 6). The PRF group showed significantly less width loss than the control group at 2 months (*I*^2^ = 76%; *p* = 0.03; MD = −0.62; 95% CI = −1.18 to −0.05) (22, 23). However, no significant differences were observed at 3 months (*I*^2^ = 86%; *p* = 0.06; MD = −0.77; 95% CI = −1.56 to −0.0.03) (13–16, 19, 20), 4 months (*I*^2^ = 87%; *p* = 0.29; MD = −0.23; 95% CI = −0.66 to −0.20) (11, 12, 18, 21, 26), or 6 months (*I*^2^ = 0%; *p* = 0.45; MD = −0.11; 95% CI = −0.41 to 0.18) (17, 24). There was no statistically significant difference in the preservation effect of PRF on alveolar bone width at different time periods (*p* = 0.26).

#### 3.4.3 Percentage of new bone formation

**Figure 7:**
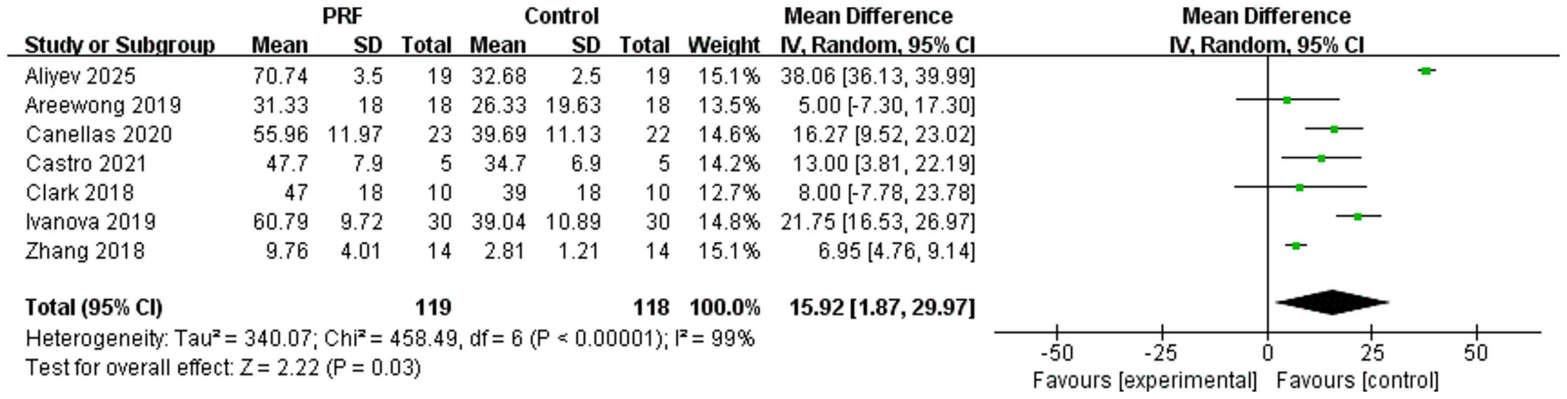
Forest plot for the meta-analysis of the percentage of new bone formation: PRF group versus control group.

Seven studies reported the percentage of new bone formation, including 119 cases in the PRF group and 118 cases in the control group (14, 15, 20, 21, 25–27). The aggregated findings demonstrated a statistically significant increase in the percentage of new bone formation in the PRF group compared to the control group. However, this result was accompanied by extreme heterogeneity (*I*^2^ = 99%) and a wide confidence interval (MD = 15.92%; 95% CI = 1.87–29.97; *p* = 0.03), indicating considerable uncertainty in the magnitude of the effect (Figure 7).

**Figure 8:**
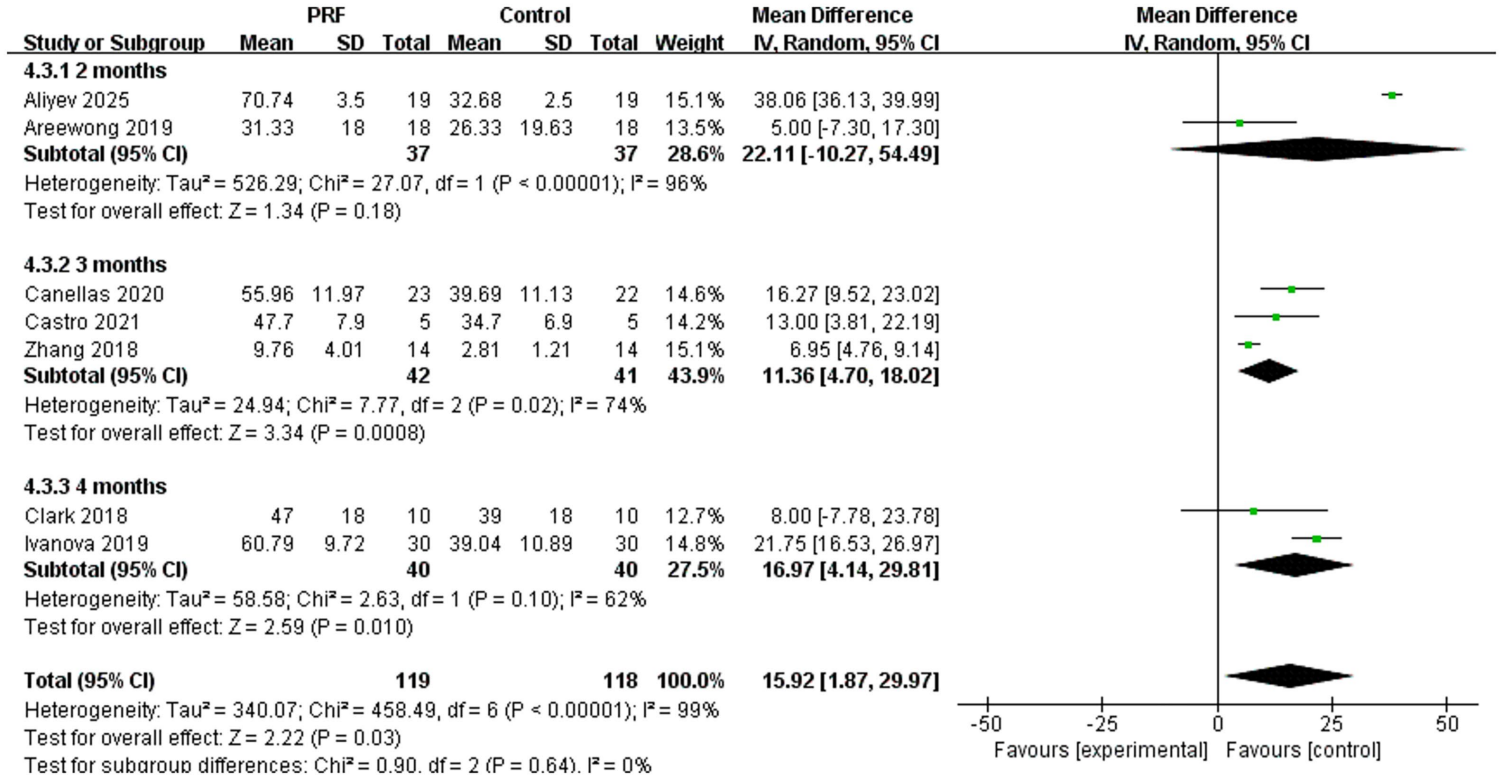
Forest plot for the subgroup meta-analysis of the percentage of new bone formation at different postoperative time points (2, 3, and 4 months): PRF group versus control group.

Subgroup analysis was performed to assess the percentage of new bone formation at various time points (Figure 8). Two studies documented the percentage of new bone formation in extraction sockets at 2 months postoperatively (25, 27). But it did not show a statistically significant difference between two groups (*I*^2^ = 96%; *p* = 0.18; MD = 22.11; 95% CI = −10.27 to −54.49). Three studies reported the percentage of new bone formation at 3 months (14, 15, 20). The findings indicated that the PRF group exhibited a markedly greater percentage of new bone formation compared to the control group, supported by a highly statistically significant overall effect test (*I*^2^ = 74%; *p* = 0.0008; MD = 11.36; 95% CI = 4.70–18.02). The percentage of new bone formation at 4 months after surgery was reported in two trials (21, 26). Results indicated that new bone formation was much higher in the PRF group than in the control group (*I*^2^ = 62%; *p* = 0.010; MD = 16.97; 95% CI = 4.14–29.81). There was no statistically significant difference in the preservation effect of PRF on new bone formation at different time periods (*p* = 0.64).

#### 3.4.4 Subgroup analysis results of PRF preparation protocol

Among these seventeen studies, one used the low-speed protocol (1,300 rpm, 14 min) (26), twelve used the standard L-PRF protocol (2,700 rpm, 12 min) (11–20, 22, 27), and four used the high-speed protocol (3,000 rpm, 10 min) (21, 23–25). Since only one study used the low-speed protocol to prepare PRF, the included data were limited, so the study did not perform a subgroup analysis on the effect of low-speed protocol.

**Figure 9:**
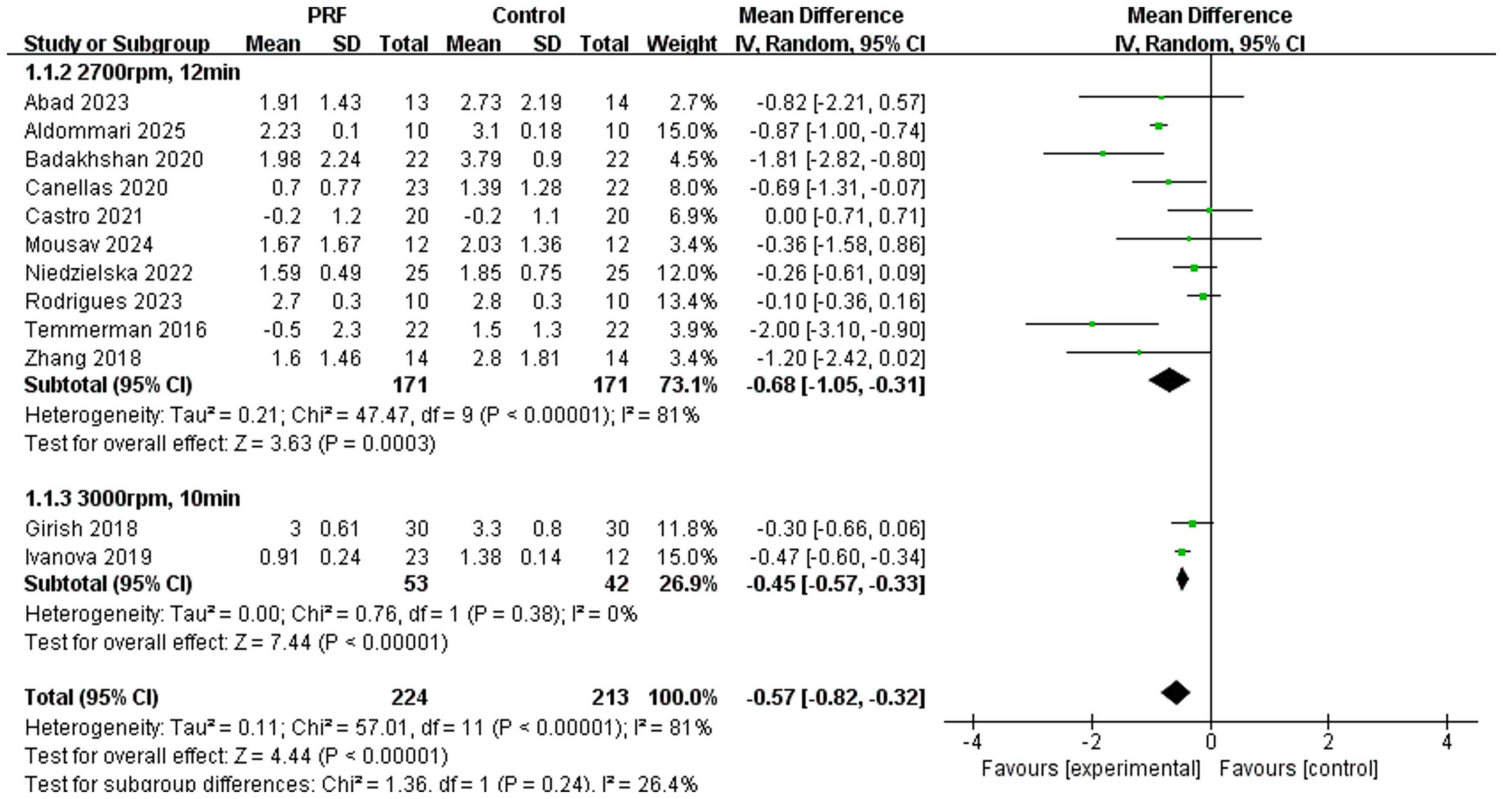
Forest plot for the subgroup meta-analysis of alveolar ridge height reduction stratified by PRF preparation protocol: PRF group versus control group.

Alveolar ridge height preservation (Figure 9): The subgroup meta-analysis revealed that both standard L-PRF and high-speed PRF protocols significantly reduced alveolar ridge height loss compared to spontaneous healing, with pooled mean differences of −0.68 mm (95% CI = −1.05 to −0.31) and −0.45 mm (95% CI = −0.57 to −0.33), respectively. While the high-speed protocol demonstrated consistent results across studies with no heterogeneity (*I*^2^ = 0%), the standard L-PRF subgroup exhibited considerable variability (*I*^2^ = 81%) and included several studies with more pronounced preservation effects (13, 19). Overall, PRF application, irrespective of centrifugation parameters, was associated with a statistically significant reduction in ridge height resorption (MD = −0.57 mm; 95% CI = −0.82 to −0.32), though the magnitude of benefit appeared more variable under standard preparation conditions. There was no significant difference in the effect of the two PRF preparation methods on the preservation of alveolar ridge height (*p* = 0.24).

**Figure 10:**
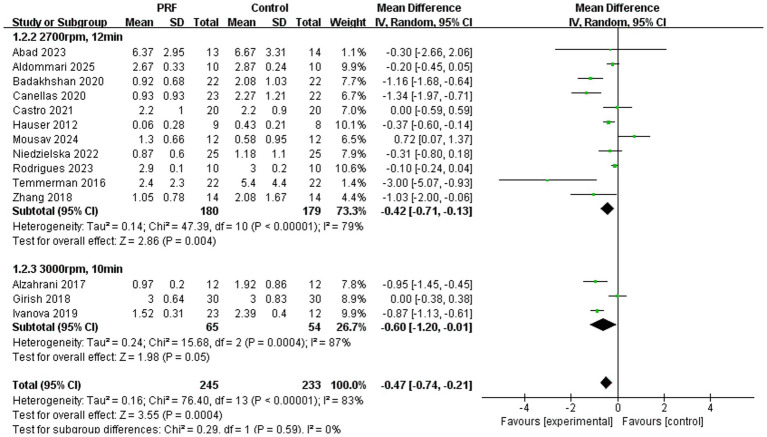
Forest plot for the subgroup meta-analysis of alveolar ridge width reduction stratified by PRF preparation protocol: PRF group versus control group.

Alveolar ridge width preservation (Figure 10): The subgroup meta-analysis for alveolar ridge width preservation demonstrated differential outcomes based on PRF preparation protocols, with no significant statistical difference between them (*p* = 0.59). The standard L-PRF protocol showed a statistically significant overall effect in reducing ridge width loss compared to spontaneous healing (*p* = 0.004), albeit with substantial heterogeneity (*I*^2^ = 79%). In contrast, the high-speed PRF protocol exhibited only borderline significance (*p* = 0.05) and markedly higher heterogeneity (*I*^2^ = 87%). Importantly, the absence of significant subgroup differences suggests that centrifugation parameters may not be the primary determinant of PRF’s effectiveness for ridge width preservation, with the observed heterogeneity likely attributable to other clinical variables.

**Figure 11:**
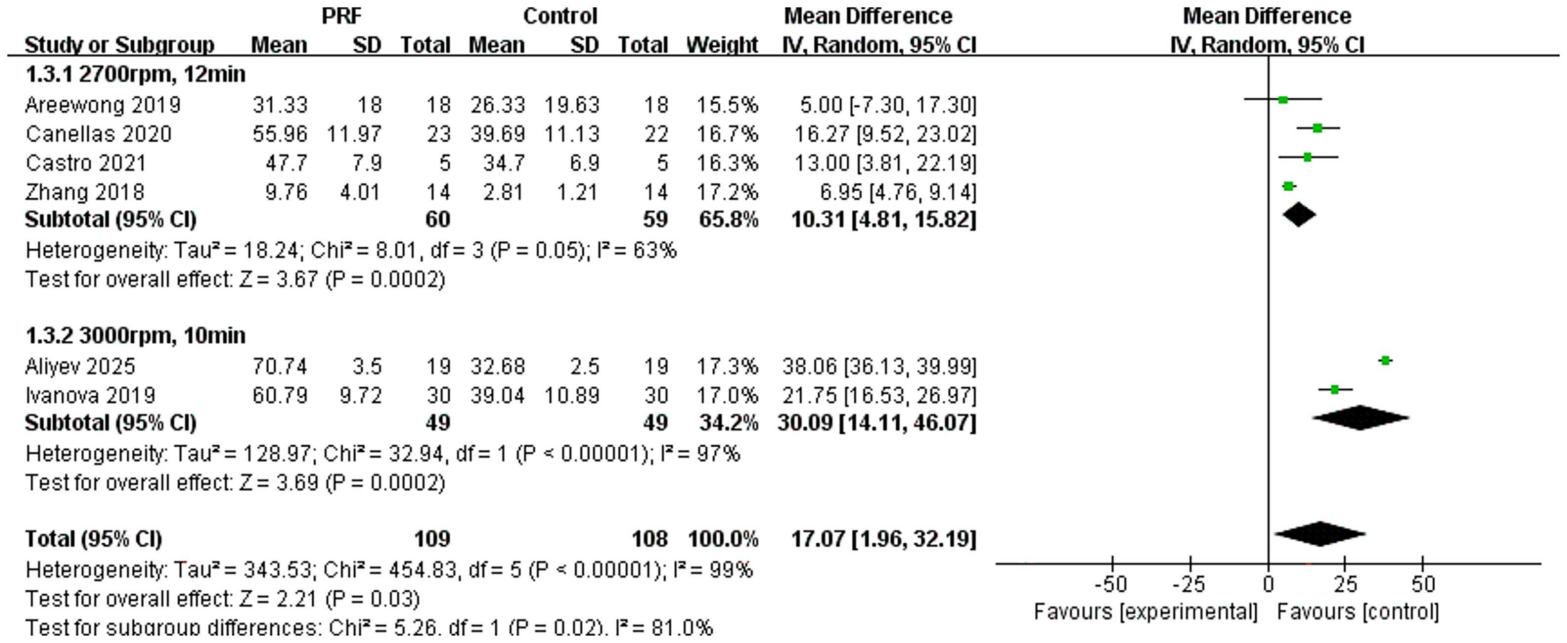
Forest plot for the subgroup meta-analysis of the percentage of new bone formation stratified by PRF preparation protocol: PRF group versus control group.

New bone formation percentage (Figure 11): The subgroup meta-analysis for new bone formation percentage demonstrated statistically significant protocol-based differences (*p* = 0.02). The subgroup utilizing the standard L-PRF protocol showed a significant increase in new bone formation (*p* = 0.0002) with moderate heterogeneity (*I*^2^ = 63%). In contrast, the high-speed PRF protocol exhibited a substantially greater effect size and a markedly higher percentage of new bone formation (*p* = 0.0002), albeit with extreme heterogeneity (*I*^2^ = 97%). However, the extremely high total heterogeneity (*I*^2^ = 99%) and the significant subgroup differences indicate that the centrifugation protocol is a critical factor influencing this outcome, with high-speed preparation appearing superior for promoting new bone formation.

### 3.5 Analysis of publication bias

**Figure 12:**
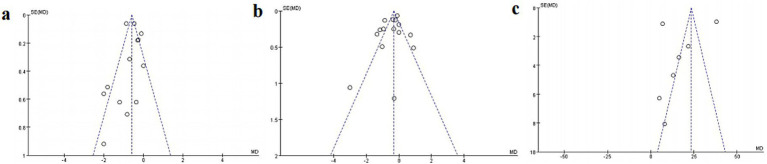
Funnel plots for assessing publication bias: **(a)** alveolar ridge height loss, **(b)** alveolar ridge width loss, **(c)** percentage of new bone formation.

The funnel plot, constructed using alveolar ridge height, width, and the percentage of new bone formation as metrics, showed that all study points fell within the inverted funnel boundaries, indicating an approximately symmetrical distribution (Figure 12). Egger’s test further confirmed the absence of significant publication bias across all outcome measures, with *p*-values of 0.227 for alveolar ridge height (Table 2), 0.758 for width (Table 3), and 0.544 for the percentage of new bone formation (Table 4). These results collectively suggest that the meta-analysis findings are robust and not substantially influenced by publication bias.

**Table 2:** Egger’s test using the amount of alveolar ridge height reduction as an indicator.

Std_Eff	Coefficient	Std. err.	*t*	*p* > |*t*|	95% CI	
Slope	−0.1268919	0.250996	−0.51	0.621	−0.6652248	0.4114409
Bias	−1.810811	1.432202	−1.26	0.227	−4.882579	1.260956

**Table 3:** Egger’s test using the amount of alveolar ridge width reduction as an indicator.

Std_Eff	Coefficient	Std. err.	*t*	*p* > |*t*|	95% CI	
Slope	−0.6032211	0.1512517	−3.99	0.003	−0.9453763	−0.2610659
Bias	−0.3420392	1.076711	−0.32	0.758	−2.09365	2.777729

**Table 4:** Egger’s test using the percentage of new bone formation as an indicator.

Std_Eff	Coefficient	Std. err.	*t*	*p* > |*t*|	95% CI	
Slope	28.7929	10.32202	2.79	0.038	2.2593	55.32649
Bias	−3.724777	5.721194	−0.65	0.544	−18.43157	10.98202

### 3.6 GRADE assessment results

The certainty of the evidence for the three primary outcomes, as assessed by the GRADE methodology, is summarized in Table 5. In the GRADE Summary of Findings table, the baseline values under “Risk with Spontaneous Healing” are standardized to 0 mm or 0% to facilitate the calculation and presentation of the MD. These values serve as reference placeholders, not as actual clinical measurements. In clinical practice, spontaneous healing typically results in a loss of alveolar ridge height of 1.0–1.5 mm and a loss of width of 3.0 to 4.5 mm within 6 months post-extraction (2, 3). Similarly, new bone formation in extraction sockets varies depending on the healing phase. The MD values presented reflect the estimated additional benefit or reduction attributable to PRF compared to spontaneous healing.

**Table 5:** Summary of findings: PRF compared to spontaneous healing for alveolar ridge preservation.

Outcomes	Anticipated absolute effects (95% CI)		Number of participants (studies)	Certainty of the evidence (GRADE)	Comments
	Risk with spontaneous healing	Risk with PRF			
Alveolar ridge height reduction	The mean height reduction was 0 mm^a^	MD 0.60 mm lower (1.85 lower to 0.34 lower)	457 (13 RCTs)	Moderate^b^	PRF likely reduces height loss
Alveolar ridge width reduction	The mean width reduction was 0 mm^a^	MD 0.42 mm lower (0.68 lower to 0.16 lower)	498 (15 RCTs)	Low^c^	PRF may reduce width loss
Percentage of new bone formation	The mean new bone formation was 0%^a^	MD 15.92% higher (1.87% higher to 29.97% higher)	237 (7 RCTs)	Low^d^	PRF may improve new bone formation, but the true effect could be substantially different

a Baseline values (0 mm/0%) are reference placeholders used to calculate MD.

b Downgraded for inconsistency (serious): considerable heterogeneity was observed (*I*^2^ = 88%).

c Downgraded for inconsistency (serious) and imprecision (serious): considerable heterogeneity was observed (*I*^2^ = 91%). The confidence interval is wide and includes a range of values from a small to a moderate effect.

d Downgraded for inconsistency (serious) and imprecision (serious): substantial heterogeneity was observed (*I*^2^ = 99%). The confidence interval is very wide and imprecise, encompassing values from a negligible to a very large increase.

Alveolar ridge height reduction: The certainty of evidence was downgraded by one level to moderate. The downgrading was solely due to serious inconsistency (considerable and unexplained heterogeneity, *I*^2^ = 80%). The possibility of bias was not regarded as serious enough to warrant downgrading for the following reasons: (1) the core outcomes (ridge height or width on CBCT) are objective, quantitative measurements; (2) outcome assessor blinding was reported in most studies, protecting against detection bias; (3) performance bias, while present, is unlikely to systematically alter the measurement of radiographic bone dimensions. The risk of bias was not considered serious enough to warrant downgrading, as the main concerns were in performance bias, which is often unavoidable for this type of intervention and is not expected to affect the objective significantly, radiographic outcome of ridge height.

Alveolar ridge width reduction: The certainty of evidence was downgraded by two levels to low. The downgrading was due to serious inconsistency (considerable heterogeneity, *I*^2^ = 83%) and imprecision (the confidence interval was wide, and some subgroup analyses showed non-significant results). Similar to height reduction, the risk of bias was not downgraded for this objective outcome.

Percentage of new bone formation: The certainty of evidence was downgraded by two levels to low. The downgrading was due to serious inconsistency (substantial heterogeneity, *I*^2^ = 99%) and serious imprecision (very wide confidence intervals that include both negligible and substantial clinical benefits). We did not downgrade for publication bias, as the funnel plot appeared roughly symmetrical and Egger’s test was not statistically significant (*p* = 0.544). However, it is important to note that the extreme heterogeneity and small number of studies limit the power of these tests to reliably detect publication bias.

## 4 Discussion

This systematic review and meta-analysis consolidates current evidence to assess the efficacy of PRF as a standalone grafting material in alveolar ridge preservation, with a specific focus on elucidating the temporal dynamics of its effects through stratified analysis. While our pooled analyses showed statistically significant benefits for PRF in several outcomes, the high to extreme heterogeneity across all primary measures necessitates a cautious interpretation.

The findings indicate that, in comparison to spontaneous healing of extraction sockets, the application of PRF is associated with a reduction in early-to-mid-term postoperative loss of alveolar ridge dimensions and an improvement in new bone formation within extraction sockets. The PRF group exhibited significantly better preservation of alveolar ridge height compared to the control group at 3, 4, and 6 months postoperatively (MD = −0.95 mm, −0.57 mm, −0.28 mm). The nearly linear trajectory of advantage suggests that PRF consistently and effectively mitigates vertical alveolar ridge resorption. A change in bone size of more than 0.5 mm is often seen as clinically significant in radiographic assessments because it is larger than the normal measurement error of serial CBCT and may affect prosthetic planning and surgical outcomes (28). In this context, the benefit of preserving ridge height observed at 3 months (MD = −0.95 mm) and 4 months (MD = −0.57 mm) exceeds this 0.5 mm threshold, suggesting that PRF application can provide a clinically relevant reduction in post-extraction vertical bone loss during the critical early- to mid-term healing phase. This level of preservation may be enough to keep the bone level above an important anatomical landmark or to give the implant enough height without the need for more vertical augmentation. Conversely, the reduction at 6 months (MD = −0.28 mm), while statistically significant, falls below this conventional clinical threshold, indicating that the absolute benefit of height preservation may diminish over time and its standalone clinical impact in the later healing stage might be limited. This temporal pattern points to PRF’s early bioactive role in stabilizing the socket.

The preservation effect of PRF on alveolar ridge width also demonstrated a significant time-dependent characteristic. The PRF group exhibited significant width preservation benefits at 2 months post-surgery (MD = −0.62 mm); however, these benefits lacked statistical significance at 3, 4, and 6 months. It can be attributed to the dual mechanism of physical barrier action and bioactive release in PRF. In the initial stages of healing, the three-dimensional fibrin network created by PRF serves as a flexible biological scaffold. The blood clot is stabilized by this structure, which also prevents the collapse of soft tissues and buccal mucosal pressure. Additionally, it creates space for bone regeneration, which is essential for the early maintenance of width (5, 8). As a degradable biomaterial, the physical support strength of PRF decreases over time, resulting in a natural reduction of its spatial maintenance capacity. Such behavior indicates that the primary advantage of PRF may not be its ability to create a lasting physical barrier akin to artificial bone substitutes but instead its remarkable biological activation properties. It elucidates the absence of variation in alveolar bone width between the two groups throughout the late healing phase. Nevertheless, the early width preservation may still hold clinical value by helping to maintain the gingival contour and soft tissue architecture during the critical early healing phase, which can influence the final prosthetic outcome.

Beyond the evidence provided by radiography, our study provided further evidence that confirmed the fundamental benefit of PRF in maximizing bone regeneration through histological evidence. The PRF group exhibited a significantly higher percentage of new bone formation at 3 and 4 months post-surgery compared to the control group. This finding demonstrates that PRF not only postpones bone resorption but also actively facilitates the bone regeneration process with enhanced quality. This intrinsic bioactivity underpins the observed benefits in new bone formation and early dimensional stabilization.

From a clinical perspective, PRF alone may be a sufficient and advantageous option for ridge preservation in sockets with intact walls and moderate initial dimensions, where the primary goal is to mitigate physiologic resorption and enhance the quality of regenerated bone without the need for exogenous graft materials. However, in cases of significant buccal bone dehiscence, large multi-rooted extraction sockets, or when substantial ridge augmentation beyond the native socket confines is required, PRF alone is likely insufficient. In such scenarios, PRF may serve best as an adjunct to structured bone grafts or barrier membranes, leveraging its bioactive properties to improve graft integration and soft tissue healing.

The results of our research are in line with the findings of a number of previously published systematic reviews. Alrayyes et al. (29) conducted a meta-analysis that concluded PRF is an effective material for alveolar ridge preservation, successfully maintaining bone width, height, and density after tooth extraction. Arora et al. (30) also endorsed the use of PRF in alveolar ridge reconstruction and guided bone regeneration, evidencing its effectiveness in enhancing ridge width, decreasing graft resorption rates, and mitigating postoperative discomfort. However, prior research primarily integrated PRF with various bone graft materials. Our study focused solely on PRF as a singular graft material for alveolar ridge preservation, eliminating the confounding effects of other materials to more accurately evaluate the intrinsic efficacy of PRF. We performed longitudinal subgroup analyses of alveolar ridge dimensional changes and new bone formation at multiple time points (2, 3, 4, and 6 months) for the first time. This approach elucidated the trajectory of PRF effects throughout various healing phases, offering precise guidance for the selection of optimal observation and intervention timings in clinical practice.

Our study revealed moderate to high heterogeneity in various outcome measures, including alveolar ridge height (*I*^2^ = 80%), width (*I*^2^ = 83%), and new bone formation (*I*^2^ = 99%), which may be due to several factors. Discrepancies in PRF preparation methods among the studies, including variations in type and centrifugation parameters, may have resulted in differences in how growth factors are released and in fibrin structure, subsequently affecting biological effects. Our findings indicate that centrifugation parameters may differentially influence specific clinical outcomes. For alveolar ridge height preservation, both standard L-PRF and high-speed protocols demonstrated significant benefits, with no statistically significant difference between them (*p* = 0.24). In contrast, the preparation protocol appeared to be a more critical factor for new bone formation. High-speed centrifugation was associated with a substantially greater increase in new bone percentage compared to the standard L-PRF protocol (*p* = 0.02). This is plausibly attributed to the higher relative yield of platelets and concentrated growth factors within the fibrin clot under higher g-forces, which may enhance the osteoinductive and osteoconductive signals during the early healing phase. Interestingly, for alveolar ridge width preservation, no significant differences were found between preparation protocols, implying that the early spatial maintenance effect might be more dependent on the physical presence of the fibrin scaffold itself rather than specific centrifugation parameters. For clinicians prioritizing maximal bone regeneration quality, high-speed protocols might be preferable. When the objective is primarily to counteract dimensional collapse, the choice between standard and alternative protocols may depend on other clinical considerations, as our analysis did not show a clear superiority of one protocol over the other for ridge preservation. Future RCTs should not only specify centrifugation speed and time in detail but also consider reporting the resulting fibrin architecture and growth factor composition to better correlate protocol variations with clinical and histological outcomes.

Furthermore, variations in extraction sites, such as anterior versus posterior teeth and single versus multiple roots, along with initial bone conditions across studies, may differentially impact healing responses and the efficacy of PRF. Assessment methods varied, with certain studies utilizing CBCT measurements, while others depended on conventional imaging or model analysis. Such methodological variations may introduce measurement bias. Surgical details and postoperative management factors, including the use of barrier membranes, suturing techniques, postoperative medication, and patient compliance, may also contribute to increased clinical heterogeneity. This study was unable to perform subgroup analyses or meta-regression analyses to confirm potential sources of heterogeneity due to insufficient reporting of pertinent data in the original literature. Furthermore, the majority of the included studies had follow-up periods ranging from 3 to 6 months. As a consequence, there was an insufficient amount of high-quality evidence regarding the long-term stability of the alveolar ridge following PRF therapy, as well as the survival and success rates of subsequent implants. The consequence of this high heterogeneity is that while a statistically significant pooled effect may be calculated, our confidence in a single, precise estimate of PRF’s efficacy for all patients and settings is low. This issue directly informs the downgrading of evidence certainty in our GRADE assessment. It is important to note that several included studies used a split-mouth design (15, 19). Ideally, meta-analyses of such studies should use paired data or employ statistical methods that account for within-patient correlation. However, due to insufficient reporting of paired outcomes in the original articles and the limited number of split-mouth studies, we utilized the group-level summary statistics to perform an analysis. These factors may have led to an overestimation of the precision of the pooled estimates. Future primary studies with split-mouth designs should report appropriate paired statistics to facilitate more accurate meta-analytic synthesis.

The application of the GRADE framework illustrates the importance of a measured interpretation of our results. The moderate-certainty evidence for decreasing alveolar ridge height offers a fairly dependable foundation for clinical evaluation. However, the low-certainty evidence for ridge width preservation and new bone formation indicates that these findings should be viewed as preliminary and suggestive rather than conclusive. The high heterogeneity underlying these ratings signals that the true effect of PRF may vary considerably across different clinical settings and techniques. Therefore, the current evidence, while promising, is not sufficient to form a strong and definitive conclusion. Future high-quality, standardized RCTs are likely to have an important impact on our confidence in the estimate of effect and may change the estimate itself.

## 5 Conclusion

Given the statistical findings alongside the notable heterogeneity and the moderate to low certainty of evidence graded by GRADE, the clinical implications of this meta-analysis should be viewed as suggestive rather than definitive. Synthesizing the available RCTs, this meta-analysis found statistically significant pooled effects suggesting that PRF may reduce post-extraction alveolar ridge height loss (moderate-certainty evidence) and might reduce width loss and improve new bone formation (low-certainty evidence). Importantly, the effect of PRF, particularly on new bone formation, may be modulated by its preparation protocol, with high-speed centrifugation showing a potentially greater osteogenic benefit. As a safe autologous biomaterial, PRF shows promise in alveolar ridge preservation. However, the low certainty for key outcomes and the protocol-dependent nature of its effects indicate that future high-quality, standardized randomized trials, which precisely report and control preparation methods, are needed to strengthen the evidence base and provide more precise, clinically actionable guidance.

## Data Availability

The original contributions presented in the study are included in the article/Supplementary material, further inquiries can be directed to the corresponding author.
