# Supplementary material for: The efficacy of platelet-rich fibrin in alveolar ridge preservation: a systematic review and meta-analysis of randomized controlled trials
Source: Front Med (Lausanne). 2026 Feb 19;13:1753189. doi: 10.3389/fmed.2026.1753189 (PMC12960125; doi:10.3389/fmed.2026.1753189)
Supplement: Supplementary file 1 [file Supplementary_file_1.docx]

Supplementary Material

Tables S1. Search strategies

Table S2. Key characteristics of studies

Table S3. PRISMA Check-list

| **Tables S1. Search strategies** | | |
| --- | --- | --- |
| **Table S1A. Search strategies (Pubmed**） | | |
| **Search** | **Query** | **Results** |
| 1 | "Platelet-Rich Fibrin"[Mesh] | 1,363 |
| 2 | ((((((((((Platelet-Rich Fibrin[Title/Abstract]) OR (Fibrin, Platelet-Rich[Title/Abstract])) OR (Platelet Rich Fibrin[Title/Abstract])) OR (Leukocyte-[Title/Abstract] AND Platelet-Rich Fibrin[Title/Abstract])) OR (Leukocyte[Title/Abstract] AND Platelet Rich Fibrin[Title/Abstract])) OR (L-PRF[Title/Abstract])) OR (leukocyte-PRF[Title/Abstract])) OR (P-PRF[Title/Abstract])) OR (pure plaletet-rich fibrin[Title/Abstract])) OR (pure PRF[Title/Abstract])) OR (thrombocyte-rich fibrin[Title/Abstract]) | 3,154 |
| 3 | ("Platelet-Rich Fibrin"[Mesh]) OR (((((((((((Platelet-Rich Fibrin[Title/Abstract]) OR (Fibrin, Platelet-Rich[Title/Abstract])) OR (Platelet Rich Fibrin[Title/Abstract])) OR (Leukocyte-[Title/Abstract] AND Platelet-Rich Fibrin[Title/Abstract])) OR (Leukocyte[Title/Abstract] AND Platelet Rich Fibrin[Title/Abstract])) OR (L-PRF[Title/Abstract])) OR (leukocyte-PRF[Title/Abstract])) OR (P-PRF[Title/Abstract])) OR (pure plaletet-rich fibrin[Title/Abstract])) OR (pure PRF[Title/Abstract])) OR (thrombocyte-rich fibrin[Title/Abstract])) | 3,180 |
| 4 | Socket Preservation[MeSH Terms] | 206 |
| 5 | (((((Socket Preservation[Title/Abstract]) OR (Ridge Preservation[Title/Abstract])) OR (Alveolar Ridge Preservation[Title/Abstract])) OR (Extraction Site Preservation[Title/Abstract])) OR (Socket Grafting[Title/Abstract])) OR (Post-extraction Grafting[Title/Abstract]) | 1,467 |
| 6 | (Socket Preservation[MeSH Terms]) OR ((((((Socket Preservation[Title/Abstract]) OR (Ridge Preservation[Title/Abstract])) OR (Alveolar Ridge Preservation[Title/Abstract])) OR (Extraction Site Preservation[Title/Abstract])) OR (Socket Grafting[Title/Abstract])) OR (Post-extraction Grafting[Title/Abstract])) | 1,656 |
| 7 | (("Platelet-Rich Fibrin"[Mesh]) OR (((((((((((Platelet-Rich Fibrin[Title/Abstract]) OR (Fibrin, Platelet-Rich[Title/Abstract])) OR (Platelet Rich Fibrin[Title/Abstract])) OR (Leukocyte-[Title/Abstract] AND Platelet-Rich Fibrin[Title/Abstract])) OR (Leukocyte[Title/Abstract] AND Platelet Rich Fibrin[Title/Abstract])) OR (L-PRF[Title/Abstract])) OR (leukocyte-PRF[Title/Abstract])) OR (P-PRF[Title/Abstract])) OR (pure plaletet-rich fibrin[Title/Abstract])) OR (pure PRF[Title/Abstract])) OR (thrombocyte-rich fibrin[Title/Abstract]))) AND ((Socket Preservation[MeSH Terms]) OR ((((((Socket Preservation[Title/Abstract]) OR (Ridge Preservation[Title/Abstract])) OR (Alveolar Ridge Preservation[Title/Abstract])) OR (Extraction Site Preservation[Title/Abstract])) OR (Socket Grafting[Title/Abstract])) OR (Post-extraction Grafting[Title/Abstract]))) | 136 |
| **Table S1B. Search strategies (Embase**） | | |
| **Search** | **Query** | **Results** |
| 1 | 'platelet-rich fibrin'/exp | 3157 |
| 2 | 'platelet-rich fibrin':ab,ti OR 'fibrin, platelet-rich':ab,ti OR 'platelet rich fibrin':ab,ti OR (leukocyte-:ab,ti AND 'platelet-rich fibrin':ab,ti) OR (leukocyte:ab,ti AND 'platelet rich fibrin':ab,ti) OR 'l prf':ab,ti OR 'leukocyte prf':ab,ti OR 'p prf':ab,ti OR 'pure plaletet-rich fibrin':ab,ti OR 'pure prf':ab,ti OR 'thrombocyte-rich fibrin':ab,ti | 3513 |
| 3 | #1 OR #2 | 3918 |
| 4 | 'socket preservation':ab,ti OR 'ridge preservation':ab,ti OR 'alveolar ridge preservation':ab,ti OR 'socket grafting':ab,ti OR 'post-extraction grafting':ab,ti | 1534 |
| 5 | #3 AND #4 | 157 |
| **Table S1C. Search strategies (Cochrane Library**） | | |
| **Search** | **Query** | **Results** |
| 1 | MeSH descriptor: [Platelet-Rich Fibrin] explode all trees | 355 |
| 2 | (Platelet-Rich Fibrin):ti,ab,kw OR (Platelet Rich Fibrin):ti,ab,kw OR (Leukocyte and Platelet-Rich Fibrin):ti,ab,kw OR (Leukocyte and Platelet Rich Fibrin):ti,ab,kw OR (Fibrin, Platelet-Rich):ti,ab,kw | 1809 |
| 3 | (L-PRF):ti,ab,kw OR (leukocyte-PRF):ti,ab,kw OR (P-PRF):ti,ab,kw OR (pure plaletet-rich fibrin):ti,ab,kw OR (pure PRF):ti,ab,kw | 278 |
| 4 | (thrombocyte-rich fibrin):ti,ab,kw | 49 |
| 5 | #1 OR #2 OR #3 OR #4 | 1891 |
| 6 | (Socket Preservation):ti,ab,kw OR (Ridge Preservation):ti,ab,kw OR (Alveolar Ridge Preservation):ti,ab,kw OR (Extraction Site Preservation):ti,ab,kw AND (Socket Grafting):ti,ab,kw | 879 |
| 7 | (Post-extraction Grafting):ti,ab,kw | 40 |
| 8 | #6 OR #7 | 891 |
| 9 | #5 AND #8 | 111 |
| **Table S1D. Search strategies (Web of Science**） | | |
| **Search** | **Query** | **Results** |
| 1 | Platelet-Rich Fibrin (Topic) OR Fibrin, Platelet-Rich (Topic) OR Platelet Rich Fibrin (Topic) OR Leukocyte- and Platelet-Rich Fibrin (Topic) OR Leukocyte and Platelet Rich Fibrin (Topic) OR L-PRF (Topic) OR leukocyte-PRF (Topic) OR P-PRF (Topic) OR pure plaletet-rich fibrin (Topic) OR thrombocyte-rich fibrin (Topic) and Preprint Citation Index (Exclude – Database) and Research Commons (Exclude – Database) | 6490 |
| 2 | Socket Preservation (Topic) OR Ridge Preservation (Topic) OR Alveolar Ridge Preservation (Topic) OR Extraction Site Preservation (Topic) OR Socket Grafting (Topic) OR Post-extraction Grafting (Topic) and Preprint Citation Index (Exclude – Database) and Research Commons (Exclude – Database) | 10570 |
| 3 | #1 AND #2 and Preprint Citation Index (Exclude – Database) and Research Commons (Exclude – Database) | 329 |

| **Table S2. Key characteristics of studies** | | | | | | | | | |
| --- | --- | --- | --- | --- | --- | --- | --- | --- | --- |
| **Author(Year)** | **Country** | **Sample Size** | **Mean Age (years)** | **Gender (M/F)** | **Outcome(s)** | **Assessment Tool(s)** | **PRF Preparation** | **Follow-Up** | **Socket Location** |
| Abad(2023) | Spain | 27 patients | 57.56±11.17 | 13/14 | •Horizontal and vertical ridge dimensional changes •Ridge contour and volumetric changes •Need for bone augmentation at implant placement •Patient-Reported Outcome Measures | •CBCT •Intraoral scans •Visual Analog Scale | 2700 rpm，12 min | 4 months | Non-molar teeth |
| Aldommari(2025) | Syria | 30 patients | 42.5 ± 7.6 | 16/14 | •Alveolar ridge width and height •Radiographic bone density •Post-operative pain level •Analgesic consumption •Soft-tissue healing •Periodontal probing depth •Gingival recession •Keratinized tissue width •Need for bone graft at implant placement | •CBCT •OnDemand3D software •Visual Analog Scale •Landry Wound Healing Index •UNC-15 periodontal probe | 2700 rpm, 12 min | 4 months | Maxillary anterior and premolar teeth |
| Alzahrani(2017) | Saudi Arabia | 24 patients | 37.8 | 9/15 | •Alveolar ridge width •Radiographic bone fill percentage | •Cast analysis with acrylic stent and digital caliper. •Computer graphic software | 3000 rpm, 10min | 1,4 and 8 weeks. | Single-tooth extraction sites |
| Badakhshan(2020) | Ecuador | 10 patients/36 sockets | NR | 7/15 | • Alveolar Height • Alveolar Width • Bone Tissue Density | CBCT | 2700 rpm,12min | 30 days and 120 days | Premolars |
| Canellas(2020) | Brazil | 48 patients | 44.8 | 21/27 | • Horizontal Bone Resorption • Vertical Bone Resorption • New Bone Volume • Histomorphometry | • CBCT • Histomorphometric Analysis | 2700 rpm,12min | 3 months | Single, non-molar teeth (Incisors, Canines, Premolars) |
| Castro(2021) | Belgium | 20 patients/60 sockets | NR | 6/15 | • Horizontal and vertical Dimensional Changes • Socket Fill • Bone Volume/Tissue Volume • Bone Microstructure | • CBCT • Histomorphometric Analysis • Micro-CT Analysis | 2700 rpm,12min | 3 months | Anterior Maxilla (Premaxilla) (Central Incisors, Lateral Incisors, Canines) |
| Girish(2018) | India | 48 patients/90 sockets | 44.4±16 | 28/62 | • Alveolar Width Loss • Alveolar Height Loss • Radiographic Bone Fill • Post-operative Pain • Swelling | • Clinical Calipers • Intraoral Radiographs (IOPA) with grid | 3000 rpm, 10min | 6 months | Anteriors, Premolars, Molars |
| Mousav(2024) | Iran | 24 patients | NR | 3/21 | •Vertical and horizontal ridge resorption •New bone formation •Bone density | •CBCT  •Histologic evaluation | 2700 rpm, 12min | 8 weeks | Single-rooted teeth (canines and premolars) |
| Niedzielska(2022) | Poland | 50 patients/100 sockets | 35.68 | 28/22 | •Soft tissue healing •Alveolar ridge width and height change •Bone density / Grayscale Value | •Clinical caliper measurements •CBCT •Author-defined Assessment Rate of Soft Tissues Healing (ARSTH) •Visual Analogue Scale (VAS) for pain | 2700rpm,12min | 10 days and 6 months | Molars, Premolars, Canines and Incisors |
| Rodrigues(2023) | Brazil | 40 patients | 41.87±11.48 | 16/24 | •Alveolar ridge height and width loss  •Assessed via CBCT and dental casts | •CBCT •Dental casts  •Dolphin Imaging 11.5 software | 2700rpm,12min | 7 and 120 days | Anterior maxilla (incisors, canines, and bicuspids) |
| Temmerman(2016) | Belgium | 22 patients/44 sockets | 54±11 | 15/7 | •Horizontal and vertical ridge dimensional changes •Socket fill •Postoperative pain | •CBCT •Superimposition of DICOM data using volumetric imaging software •Dutch version of the McGill Pain •Questionnaire (MPQ-DLV) with Visual Analogue Scale (VAS) | 2700rpm,12min | 3 months | incisors, canines, premolars |
| Zhang(2018) | China | 28 patients | 33.9 | 14/14 | •Gingival healing •Alveolar ridge height, width, and bone mineral density •Histomorphometric evaluation of new bone formation •Postoperative pain and soft tissue healing | •CBCT •Scanning electron microscopy •Light microscopy with Goldner’s trichrome staining •Clinical evaluation of gingival healing | 2700rpm, 12min | 3 months | molars |
| Clark(2018) | United States | 40 patients | 58 | 18/22 | •Alveolar ridge height and width •Percentage of vital bone formation •Bone mineral density | •Custom resin measurement stent with periodontal probe and calipers •Micro-CT •Histomorphometric analysis | 1300rpm, 14min | 15 weeks | Single-rooted teeth (non-molar) |
| Ivanova(2019) | Bulgaria | 90 patients | 41.60±10.95 | 45/45 | •Percentage of vital bone formation •Percentage of connective tissue •Percentage of residual graft particles/immature bone | •Histomorphometric analysis •ImageJ software for analysis •CBCT | 3000rpm, 10min | 4 months | Front teeth, premolars, molars |
| Hauser(2012) | Switzerland | 23 patients | 47 | 9/14 | •Bone microarchitecture  •Intrinsic bone tissue quality •Alveolar crest width preservation •Vertical bone height changes •Clinical soft tissue healing | •Micro-computed tomography •Nanoindentation test •Caliper clamp •Superimposable periapical radiographs •Clinical evaluation | 2700rpm,12min | 8 weeks | premolars |
| Aliyev(2025) | Turkey | 57 patients | 45±5.6 | 30/27 | •Percentage of new bone formation •Expression of bone regenerative markers  •Postoperative pain •Gingival swelling •Membrane visibility •Healing | •Histomorphometric analysis •Immunohistochemistry •Visual Analog Scale •Clinical observation | 3000 rpm,10min | 8 weeks | Mandibular Anterior Teeth |
| Areewong(2019) | Thailand | 33 patients/36 sockets | 50.67 | 15/21 | New bone formation ratio | •Histomorphometric analysis •Image J Software | 2700rpm,12min | 2 months | Single-rooted Premolars and Maxillary Anterior Teeth |

**Table S3. PRISMA Check-list**

| **Section and Topic** | **Item #** | **Checklist item** | **Location where item is reported** |
| --- | --- | --- | --- |
| **TITLE** | | |  |
| Title | 1 | Identify the report as a systematic review. | P1 |
| **ABSTRACT** | | |  |
| Abstract | 2 | See the PRISMA 2020 for Abstracts checklist. | P1, L10-35 |
| **INTRODUCTION** | | |  |
| Rationale | 3 | Describe the rationale for the review in the context of existing knowledge. | P2, L60-78 |
| Objectives | 4 | Provide an explicit statement of the objective(s) or question(s) the review addresses. | P2, L70-78 |
| **METHODS** | | |  |
| Eligibility criteria | 5 | Specify the inclusion and exclusion criteria for the review and how studies were grouped for the syntheses. | P3, L93-102 |
| Information sources | 6 | Specify all databases, registers, websites, organisations, reference lists and other sources searched or consulted to identify studies. Specify the date when each source was last searched or consulted. | P3,L85-86 |
| Search strategy | 7 | Present the full search strategies for all databases, registers and websites, including any filters and limits used. | P3, L85-91 |
| Selection process | 8 | Specify the methods used to decide whether a study met the inclusion criteria of the review, including how many reviewers screened each record and each report retrieved, whether they worked independently, and if applicable, details of automation tools used in the process. | P3, L104-107 |
| Data collection process | 9 | Specify the methods used to collect data from reports, including how many reviewers collected data from each report, whether they worked independently, any processes for obtaining or confirming data from study investigators, and if applicable, details of automation tools used in the process. | P3, L104-107 |
| Data items | 10a | List and define all outcomes for which data were sought. Specify whether all results that were compatible with each outcome domain in each study were sought (e.g. for all measures, time points, analyses), and if not, the methods used to decide which results to collect. | P3, L97 |
|  | 10b | List and define all other variables for which data were sought (e.g. participant and intervention characteristics, funding sources). Describe any assumptions made about any missing or unclear information. | P3, L104-107 |
| Study risk of bias assessment | 11 | Specify the methods used to assess risk of bias in the included studies, including details of the tool(s) used, how many reviewers assessed each study and whether they worked independently, and if applicable, details of automation tools used in the process. | P3, L108-112 |
| Effect measures | 12 | Specify for each outcome the effect measure(s) (e.g. risk ratio, mean difference) used in the synthesis or presentation of results. | P3,L14-119 |
| Synthesis methods | 13a | Describe the processes used to decide which studies were eligible for each synthesis (e.g. tabulating the study intervention characteristics and comparing against the planned groups for each synthesis (item #5)). | P2,L114-127；L93-105 |
|  | 13b | Describe any methods required to prepare the data for presentation or synthesis, such as handling of missing summary statistics, or data conversions. | P4,L123-127 |
|  | 13c | Describe any methods used to tabulate or visually display results of individual studies and syntheses. | P4,L146 |
|  | 13d | Describe any methods used to synthesize results and provide a rationale for the choice(s). If meta-analysis was performed, describe the model(s), method(s) to identify the presence and extent of statistical heterogeneity, and software package(s) used. | P3,L114-119 |
|  | 13e | Describe any methods used to explore possible causes of heterogeneity among study results (e.g. subgroup analysis, meta-regression). | P4,L120-127 |
|  | 13f | Describe any sensitivity analyses conducted to assess robustness of the synthesized results. | Not mentioned |
| Reporting bias assessment | 14 | Describe any methods used to assess risk of bias due to missing results in a synthesis (arising from reporting biases). | P4,L118-119 |
| Certainty assessment | 15 | Describe any methods used to assess certainty (or confidence) in the body of evidence for an outcome. | P4,L128-137 |
| **RESULTS** | | |  |
| Study selection | 16a | Describe the results of the search and selection process, from the number of records identified in the search to the number of studies included in the review, ideally using a flow diagram. | P4,L140-146 |
|  | 16b | Cite studies that might appear to meet the inclusion criteria, but which were excluded, and explain why they were excluded. | P4,L140-146 |
| Study characteristics | 17 | Cite each included study and present its characteristics. | P4,L148-164 |
| Risk of bias in studies | 18 | Present assessments of risk of bias for each included study. | P5,L168-186 |
| Results of individual studies | 19 | For all outcomes, present, for each study: (a) summary statistics for each group (where appropriate) and (b) an effect estimate and its precision (e.g. confidence/credible interval), ideally using structured tables or plots. | Figure 3, 4, 5, 6, 7, 8, 9, 10, 11, Table1 |
| Results of syntheses | 20a | For each synthesis, briefly summarise the characteristics and risk of bias among contributing studies. | P4,L147-186 |
|  | 20b | Present results of all statistical syntheses conducted. If meta-analysis was done, present for each the summary estimate and its precision (e.g. confidence/credible interval) and measures of statistical heterogeneity. If comparing groups, describe the direction of the effect. | P6,L187-286 |
|  | 20c | Present results of all investigations of possible causes of heterogeneity among study results. | P7,L246-286 |
|  | 20d | Present results of all sensitivity analyses conducted to assess the robustness of the synthesized results. | Not mentioned |
| Reporting biases | 21 | Present assessments of risk of bias due to missing results (arising from reporting biases) for each synthesis assessed. | P8,L288-299 |
| Certainty of evidence | 22 | Present assessments of certainty (or confidence) in the body of evidence for each outcome assessed. | P9,L301-332 |
| **DISCUSSION** | | |  |
| Discussion | 23a | Provide a general interpretation of the results in the context of other evidence. | P10,L334-453 |
|  | 23b | Discuss any limitations of the evidence included in the review. | P11,L401-422 |
|  | 23c | Discuss any limitations of the review processes used. | P12,L435-444 |
|  | 23d | Discuss implications of the results for practice, policy, and future research. | P12,L455-466 |
| **OTHER INFORMATION** | | |  |
| Registration and protocol | 24a | Provide registration information for the review, including register name and registration number, or state that the review was not registered. | P3,L83 |
|  | 24b | Indicate where the review protocol can be accessed, or state that a protocol was not prepared. | P3,L80-83 |
|  | 24c | Describe and explain any amendments to information provided at registration or in the protocol. | Not mentioned |
| Support | 25 | Describe sources of financial or non-financial support for the review, and the role of the funders or sponsors in the review. | P13,L475 |
| Competing interests | 26 | Declare any competing interests of review authors. | P13,L468-470 |
| Availability of data, code and other materials | 27 | Report which of the following are publicly available and where they can be found: template data collection forms; data extracted from included studies; data used for all analyses; analytic code; any other materials used in the review. | P16,L573-575 |
